# Immune response after systematic lymph node dissection in lung cancer surgery: changes of interleukin-6 level in serum, pleural lavage fluid, and lung supernatant in a dog model

**DOI:** 10.1186/1477-7819-11-270

**Published:** 2013-10-10

**Authors:** Seong Yong Park, Dae Joon Kim, Abdullah Aldohayan, Iftikhar Ahmed, Sufia Husain, Ammar Al Rikabi, Abdulazeem Aldawlatly, Omar Al Obied, Waseem Hajjar, Sami Al Nassar

**Affiliations:** 1 Department of Thoracic and Cardiovascular Surgery, Yonsei University, College of Medicine, 50 Yonsei-ro, Seodaemun-gu, Seoul 120-752, Republic of Korea; 2 Department of Surgery, King Saud University, College of Medicine, PO Box 2925, Riyadh 11461, Kingdom of Saudi Arabia; 3 Department of Histopathology, King Saud University, College of Medicine, PO Box 2925, Riyadh 11461, Kingdom of Saudi Arabia; 4 Department of Anesthesia, King Saud University, College of Medicine, PO Box 2925, Riyadh 11461, Kingdom of Saudi Arabia

**Keywords:** IL-6, Lung cancer, Mediastinal lymph node dissection

## Abstract

**Background:**

Systematic nodal dissection (SND) is regarded as a core component of lung cancer surgery. However, there has been a concern on the increased morbidity associated with SND. This study was performed to investigate whether or not SND induces significant immune response.

**Methods:**

Sixteen dogs were divided into two groups; group 1 (n = 8) underwent thoracotomy only, and group 2 (n = 8) underwent SND after thoracotomy. We compared interleukin-6 (IL-6) levels in serum, pleural lavage fluid and lung supernatant at the time of thoracotomy (T0) and at 2 h(T1) after thoracotomy (group 1) or SND (group 2). Severity of inflammation and IL-6 expression in lung tissue were evaluated in a semi-quantitative manner.

**Results:**

The operative results were comparable. IL-6 was not detected in serum in either group. IL-6 in pleural lavage fluid marginally increased from 4.75 ± 3.74 pg/mL at T0 to 19.75 ± 8.67 pg/mL at T1 in group 1 (*P* = 0.112), and from 7.75 ± 5.35 pg/mL to 17.72 ± 8.58 pg/mL in group 2 (*P* = 0.068). IL-6 in lung supernatant increased from 0.36 ± 0.14 pg/mL/mg to 1.15 ± 0.17 pg/mL/mg in group 1 (*P* = 0.003), and from 0.25 ± 0.08 pg/mL/mg to 0.82 ± 0.17 pg/mL/mg in group 2 (*P* = 0.001). However, the degree of increase in IL-6 in pleural lavage fluid and lung supernatant were not different between two groups (*P* = 0.421 and *P* = 0.448). There was no difference in severity of inflammation and IL-6 expression between groups.

**Conclusions:**

SND did not increase IL-6 in pleural lavage fluid and lung supernatant. This result suggests that SND could be routinely performed in lung cancer surgery without increasing the significant inflammatory response.

## Background

Mediastinal and hilar lymph node dissection has been a core component of lung cancer surgery since Cahan reported the first series of pulmonary lobectomies with regional lymph node dissection [1]. In 1996, the International Association for the Study of Lung Cancer (IASLC) accepted systematic nodal dissection (SND) as an integral feature of intra-thoracic cancer surgery [2]. During SND, all lymph nodes and surrounding fatty tissue within anatomic landmarks are removed, enabling accurate surgical staging. In addition, SND has the potential to provide survival benefits over systematic lymph node sampling (SLS), in which certain lymph nodes are removed to a lesser extent. However, concerns have been raised regarding increased morbidity rates associated with SND. Okada et al. reported a significantly higher morbidity rate for SND as compared to SLS, which may be attributed to increased tissue damage [3]. In contrast, several other studies failed to demonstrate differences in operative morbidity between SND and SLS [4,5]. To date, a consensus regarding the potential additional surgical injury as a result of SND has not been reached. One explanation might be that all studies included a concomitant pulmonary resection followed by SND, which prevented them from specifically attributing any observed surgical morbidity to the application of SND. In addition, the type of resection (sub-lobar resection, lobectomy, or pneumonectomy), the amount of blood loss during pulmonary resection, and the total operating time, are all significant contributors to postoperative morbidity, which may obscure the determination of differences caused by the type of lymphadenectomy.

The concentration of immune response mediators as measured in body fluids is a well-established indicator of surgical injury. Among the cytokines activated during the acute phase response after a major surgery, interleukin-6 (IL-6) has been correlated to the severity of surgical stress [6] as well as the subsequent morbidity and mortality [7,8]. After an injury, the levels of IL-6 in the circulation become detectable within 60 min, peak between 2 and 3 hours, and can persist for as long as 10 days in humans [9]. In canine models, IL-6 levels are also significantly increased following a local injury, and change faster than other acute phase proteins, such as tumor necrosis factor or C-reactive protein (CRP) [10]. The purpose of this study was to investigate whether SND carries an added risk of significant surgical injury. To achieve this, we evaluated the changes in IL-6 in serum, pleural lavage fluid, and lung supernatant in a canine thoracotomy model without performing a pulmonary resection.

## Methods

### Experimental animals

A total of 16 dogs, weighing 10.5 to 20.1 kg, with similar genetic backgrounds were used in this study. The animals were housed in cages maintained at a controlled temperature of 22 to 24°C and kept on a standard diet. The dogs were assigned group 1 (n = 8), which underwent thoracotomy only, or group 2 (n = 8), which underwent thoracotomy followed by SND. The experimental animal protocol was approved by the Animal Ethics Board, College of Medicine Research Centre, King Saud University.

### Operation

Anesthesia was induced by intramuscular injection of ketamine (4 mg/kg)/xylazine (0.4 mg/kg), after which orotracheal intubation was performed using a 7.0 to 7.5 mm endotracheal tube. A tracheostomy was performed, allowing introduction of a smaller endotracheal tube into the left main bronchus, under bronchoscopic guidance, to achieve the single-lung ventilation. The anesthesia was maintained with 0.5% of sevoflurane, and mechanical ventilation was employed with a tidal volume of 10 mL/kg and a respiratory rate of 25 to 30 breaths/min. Electrocardiography and pulse oximetry were monitored throughout the procedure.

A right lateral thoracotomy was performed after administration of antibiotics, and the intercostal space was spread using a self-retractor. As soon as the thoracotomy was completed, 5 mL of venous blood was sampled, and 100 mL of warm normal saline was injected into the pleural cavity before any further manipulation. The fluid was irrigated by hand over the visceral and parietal pleura for 1 min, and sampled. Lung tissue was harvested from the edge of the anterior segment of the right upper lobe using an endostapler. The blood and pleural lavage samples were forwarded to the immunology laboratory, and the lung tissue was sent fresh in a sealed plastic container.

For the animals in group 1, no further procedure was added, and the sampling was redone 2 hours following the start of the thoracotomy; this time frame was chosenbecause the levels of IL-6 in the circulation become detectable within 60 min, and peak between 2 and 3 hours after surgical injury [9]. In group 2, SND of pulmonary hilum and mediastinum was performed following the initial thoracotomy. With the right lung under retraction, *en bloc* resection of all the lymph nodes and surrounding fatty tissue within the anatomic landmarks (superior vena cava, trachea, bronchus, and pericardium) was carried out. After 2 hours, blood, pleural fluid lavage, and lung tissue were sampled in a manner consistent with group 1. Thus, we compared the levels of IL-6 in serum, pleural lavage and lung supernatant at the time of thoracotomy (T0) and at 2 hours (T1) after either thoracotomy (group 1) or SND (group 2). The total operation time, SND time, and the amount of blood loss were recorded. After T1, the thoracotomy was closed, and the animals were monitored for 2 weeks.

Blood and pleural lavage fluid were cooled to 4°C, centrifuged at 2,500 rpm for 10 min, transferred into sterile 1.0 mL tubes and preserved at −80°C for later analysis. Each lung tissue sample was bisected and each half was weighed. One half of the lung tissue was forwarded to the immunology laboratory for ELISA and the other was submitted for histopathological evaluation.

### Hematoxylinand eosin (H&E) staining and immunohistochemistry (IHC)

Lung tissue was fixed in 10% formalin, then processed in a Tissue-Tek® VIP®6 vacuum infiltration processor (Sakura Finetek, Tokyo, Japan). The tissue samples were embedded in paraffin, cut in 5-μm thick sections using a microtome, and either stained with H&E or mouse monoclonal IL-6 antibody (BD Bioscience, San Jose, USA) for IHC. Two histopathologists examined the slides in a double-blinded manner, and independently reported their findings.

H&E samples were evaluated for the presence and type of inflammatory cells (lymphocytes, histiocytes, macrophages and neutrophils). An assessment of inflammatory infiltration was made for specific areas of the lung tissue (subpleural, peribronchial and random location). Because there is no standardized grading system for the severity of inflammation and the degree of IL-6 staining in this model, we devised the grading system as follows; negative: less than 5 inflammatory cells per 3 high power fields (HPFs); mild: 5 to 10 inflammatory cells per 3 HPFs; moderate: 10 to 15 inflammatory cells per 3 HPFs; and severe: >15 inflammatory cells per 3 HPFs. The purpose of IHC staining was to identify the IL-6 expressing inflammatory cells. To evaluate the amount of IL-6 positive cells, a semi-quantitative scoring system was applied as follows. For the amount of positive cells: no cells stained: 0; less than 5 inflammatory cells show IL-6 staining: 1; 5 to 25%: 2; 26 to 50%: 3; 51 to 75%: 4; 76 to 100%: 5. For the signal intensity; 1: weak staining, 2: moderate staining, 3: strong staining. The maximum combined score of proportion and intensity was 8. Non-specific IL-6 positive cells such as intra-alveolar macrophages and bronchial epithelial cells were identified and disregarded.

### ELISA assay

A section of the lung tissue sample was weighed and immediately incubated with RPMI-1640 culture medium supplemented with 10% fetal calf serum in an atmosphere of 95% air and 5% CO_2_ at 37°C. After 12 hours, the supernatant was collected. The IL-6 levels were measured using the ELISA technique according to the manufacturer’s instructions (Diasource, Nivelles, Belgium) and expressed as pg IL-6 per mL of supernatant (pg/mL) derived from 1 mg lung tissue. In addition, plasma and pleural lavage fluid were subjected to ELISA using the same antibody, and those values were expressed as pg IL-6 per mL plasma or pleural lavage fluid, respectively.

### Statistical analysis

The results were expressed as mean ± standard error of the mean. The differences between the two groups were analyzed using independent *t*-test. Changes within the groups were analyzed using a paired *t*-test. All data were recorded in a database and analyzed with SPSS 20.0 software (SPSS Inc., Chicago, IL, USA). A result was considered statistically significant when the *P*value was less than 0.05. All statistical analysis were reviewed and verified by a statistician.

## Results

### Operative results

The operative results are described in Table 1. Between the two groups the body weight, anesthesia induction time, and estimated blood loss were comparable. As SND was added to the procedure in group 2, taking 11.75 ± 0.90 min, the total operating time was longer in group 2 than in group 1 (151.25 ± 2.70 vs. 162.13 ± 2.46, *P*= 0.027). During SND, an average of 3.44 ± 0.37 lymph nodes were dissected, weighting 2.88 ± 0.91 mg. There was no incidence of mortality in either group.

**Table 1:** Operative data

	**Group 1**	**Group 2**	***P***
Body weight (kg)	13.70 ± 1.02	15.18 ± 0.94	0.304
Induction time for anaesthesia (minutes)	28.50 ± 1.42	35.08 ± 3.37	0.082
Total operating time (minutes)	151.25 ± 2.70	162.13 ± 3.46	0.027
Systematic node dissection time (minutes)	N/A*	11.75 ± 0.90	N/A*
Weight of lymph nodes (mg)	N/A*	3.44 ± 0.37	N/A*
Number of lymph nodes	N/A*	2.88 ± 0.91	N/A*
Estimated blood loss (mL)	6.88 ± 0.91	32.50 ± 14.82	0.106

*N/A; not applicable.

### H&E staining

For H&E staining, samples were derived from all but one animal, and 5 to 10 inflammatory cells were observed per 3 HPFs; most inflammatory infiltrates were composed of lymphocytes, histiocytes, or interstitial macrophages. There was no difference in the degree of inflammation between the two groups, and the amount of inflammatory infiltrates was not increased by the SND procedure.

### IHC

**Figure 1:**
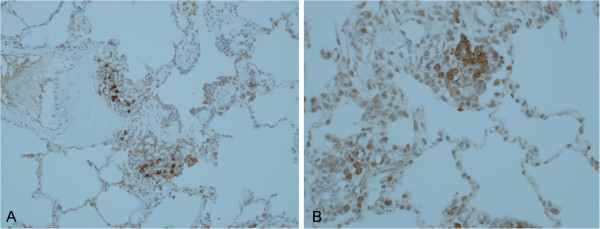
**IL-6 expression in lung tissue.** The photomicrograph shows sections from a dog lung treated with IL-6 IHC stains before (**A**, ×200) and after SND (**B**, ×400). There are aggregates of mononuclear inflammatory cells with moderately intense staining of cytoplasm and cell membrane. There was no difference in IL-6 expression score between the groups.

At T0, inflammatory cells from both groups were found to express IL-6. The expression scores were similar (3 (range: 0–4) for group 1, vs.2.5 (range: 0–4) for group 2, *P* = 0.3). The IL-6 scores at T1 (3 (range: 2–4) for group 1 and 3 (range: 2–5) for group 2) did not differ significantly (*P*= 0.3). No differences in immunohistochemical staining patterns were observed after SND in group 2 (Figure 1).

### IL-6 levels in pleural lavage fluid

**Figure 2:**
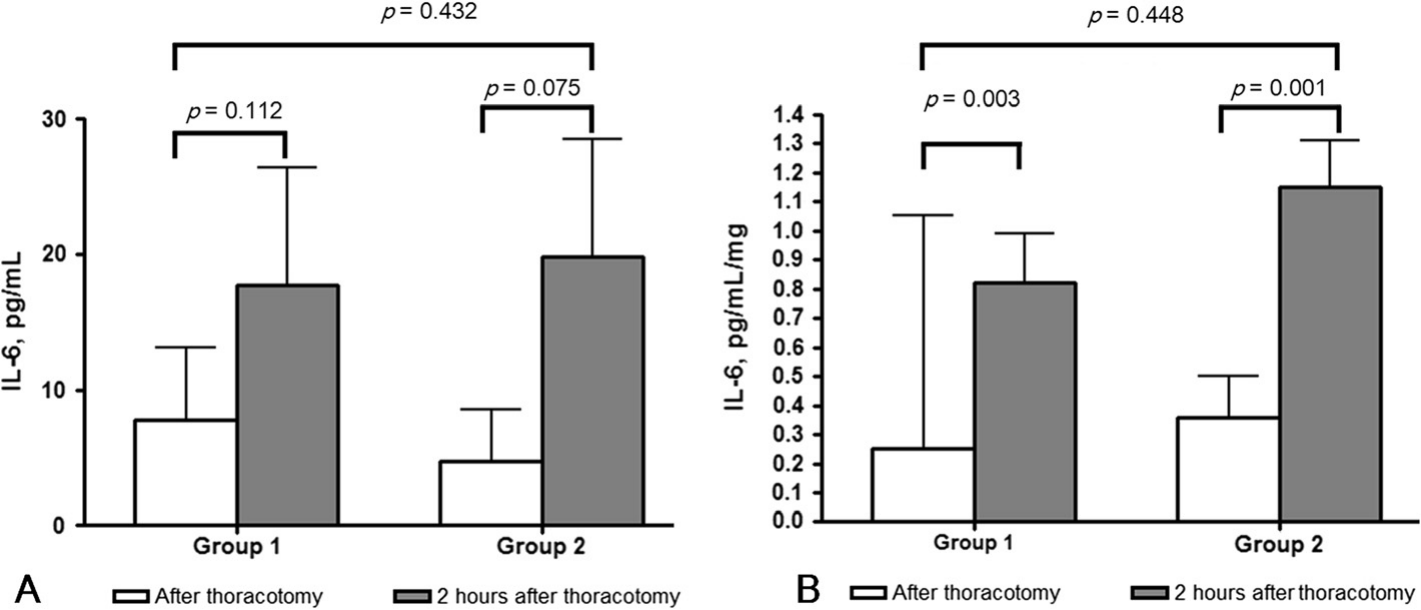
**IL-6 levels in pleural lavage fluid (A) and lung supernatant (B). (A)** In both groups, IL-6 was marginally increased 2 hours after thoracotomy, but there was no difference between group 1 and group 2 (*P*= 0.432). **(B)** IL-6 was significantly increased in both groups 2 hours after thoracotomy, but there was no difference between group 1 and group 2 (*P*= 0.448).

The difference in IL-6 levels at T0 (4.75 ± 3.74 pg/mL in group 1, and 7.75 ± 5.35 pg/mL in group 2) did not reach statistical significance (*P* = 0.721). At T1, the IL-6 levels were marginally increased (19.75 ± 8.67 pg/mL in group 1, *P* = 0.112 and 17.72 ± 8.58 pg/mL in group 2, *P*= 0.075), but there was no significant difference between the groups (*P*= 0.432, Figure 2A).

### IL-6 levels in lung supernatant and serum

In both groups the IL-6 levels increased significantly over time (group 1: 0.36 ± 0.14 pg/mL/mg at T0, 1.15 ± 0.17 pg/mL/mg at T1, *P*= 0.003, vs. group 2: 0.25 ± 0.08 pg/mL/mg at T0, 0.8 ± 0.17 pg/mL/mg at T1, *P*= 0.001). There were no significant differences between the two groups (*P*= 0.448, Figure 2B). IL-6 was not detectable in the serum in either group, even after SND was performed.

## Discussion

SND is an essential component of lung cancer surgery. It facilitates accurate staging and guidance for adjuvant therapy after resection. In 1996, SND was accepted by the IASLC as a core component of intrathoracic lung cancer staging [2]. SND is regarded as mandatory in some regions, where it is believed to increase the probability of curative therapy, hence improving the survival rate. However, a recent survey revealed that approximately 40% of surgical lung cancer patients did not undergo any lymph node dissection at the time of surgery [11].

There are several explanations for the low rate of SND, the main reasons being longer operation time and concerns about the potentially associated morbidity. Several clinical studies showed contradictory results; Okada et al. reported a higher incidence of pneumonia, atelectasis, and chylothorax after SND when compared with SLS [3], whereas other studies reported similar outcomes between the two modalities [4,5,12]. However, these reports are observational clinical studies, in which all patients underwent a pulmonary resection before or following the SND. Compared to major surgery, the effects of SND are likely negligible. For example, a patient undergoing a pneumonectomy without lymph node dissection might endure more surgical stress than a patient undergoing a pulmonary lobectomy with SND. In addition, the amount of bleeding, the effectiveness of single-lung ventilation, the fluid balance, and the total operating time all have an influence on the postoperative morbidity. These factors complicate the comparison of pulmonary surgery patients with regards to the contribution of SND to postoperative morbidity. We therefore opted for an experimental study, in which we could evaluate the surgical injuries as caused by SND while excluding any overshadowing effects caused by a concomitant pulmonary resection.

We used IL-6 as a marker for the extent and severity of surgical injury. Among cytokines activated during the acute phase response, IL-6 is significantly correlated with postoperative mortality and morbidity rates [7,8]. Recent studies have revealed that IL-6 is produced locally at the surgical site, and a variety of cells such as alveolar macrophages, lung fibroblasts, and airway epithelial cells can produce IL-6 under certain conditions [13,14]. In our canine model, IL-6 was significantly increased following local injury, which is consistent with reports detailing the IL-6 response in humans [10].

Our study showed that SND does not independently elicit a significant surgical injury. The IL-6 level was increased in pleural lavage fluid and lung supernatant in both groups regardless of the performance ofSND. Considering that IL-6 was increased at T1 in group 1, this increase seems to have been caused by factors independent of SND. For instance, single-lung ventilation might promote the production and release of IL-6 in the alveoli. A high inspiratory pressure in the contralateral lung might cause barotrauma and subsequent deterioration of the alveolar-capillary barrier [15]. In addition, single-lung ventilation-induced atelectasis and re-expansion might induce ischemia-reperfusion injury, which leads to the production of inflammatory cytokines [16]. Also, mechanical stress on the right lung, such as retraction, compression, or massaging of lung tissue, may also cause production of IL-6. Finally, the thoracotomy itself may induce surgical stress. It is well known that a lobectomy via thoracotomy causes a higher cytokine response than a lobectomy via video-assisted thoracic surgery [17]. Given that the degree of change in IL-6 levels in both groups was not significant, we conclude that SND had little effect on the production and release of IL-6. Therefore, our findings support the position that SND is a safe and feasible procedure which does not increase the surgical stress.

While IL-6 was not detected in serum in either group, it was increased in pleural lavage fluid and lung supernatant. On IHC staining of lung tissue, IL-6 was mainly expressed on lymphocytes, histiocytes, and interstitial macrophages. These findings suggest that in the very early postoperative period, these cells may play an important role in the production of IL-6. Recent studies reported that bronchial epithelial cells were responsible for IL-6 production *in vivo* and *in vitro*[18,19]. Lung tissue may be a potential source of cytokine production during thoracic surgery.

This study has some limitations. First, because we intended to analyze the ‘acute’ response following surgery, we measured the IL-6 levels only for 2 hours after the procedure. In order to document the effect of SND on postoperative morbidity, serial follow-up of IL-6 and monitoring of complications such as pneumonic infiltration on X-ray until the seventh postoperative day are needed. Given that the IL-6 level peaks within 2 hours of surgery [20], our method seems adequate to compare the degree of acute surgical injury between two groups. Second, we measured only IL-6 as an inflammatory marker because IL-6 has been correlated to the severity of surgical stress and subsequent mortality and morbidity [6-8]. Measuring other acute phase proteins such as tumor necrosis factor and CRP could offer more information on the inflammatory changes. However, we thought that IL-6 is the best indicator for this study because it is significantly increased after a local injury and is elevated faster than other acute phase proteins [10]. Third, we did not measure anti-inflammatory cytokines. The regulation of inflammation by anti-inflammatory cytokines is complicated as a number of related external factors should be considered; further, there have been no studies on the relationship between these cytokines and surgical injury in a canine model. Because IL-6 has been reported as an indicator of inflammatory status in both human and animals, we tested IL-6 levels only. Finally, even though the mean number of dissected lymph nodes after complete mediastinal lymph node dissection in group 2 was about 2.8, the paucity of dissected lymph nodes could be criticized as a limitation. However, during thoracic operations of dogs and pigs for training purposes, we observed few lymph nodes in either of these animals. Even though dissected lymph nodes in this study were small, we completely dissected lymph nodes and fat tissue at each station. In spite of these limitations, to our knowledge, this is the first study investigating whether SND carries an added risk of significant surgical injury in an animal model.

## Conclusions

In summary, SND did not independently contribute to an increase in the level of IL-6 in serum, pleural lavage fluid, and lung supernatant in a canine model of thoracotomy. These results support the routine application of SND during lung cancer surgery because it offers accurate staging and increased likelihood of curative therapy in selected patients without increasing the significant inflammatory response.

## Abbreviations

H&E: Hematoxylin & Eosin; HPF: High power field; IASLC: International Association for the Study of Lung Cancer; IHC: Immunohistochemistry; IL-6: Interleukin-6; SLS: Systemic lymph node sampling; SND: Systemic nodal dissection.

## Competing interests

The authors declare that they have no competing interests.

## Authors’ contributions

SYP performed statistical analysis and wrote the manuscript. DJK performed animal experiments and wrote and reviewed the manuscript. AA, IA, OAO, WH, SAN and AE participated in animal experiments. SH and AAR performed immunohistochemical analysis. All authors read and approved the final manuscript.
